# The utility of using fiberoptic rhinoscopy in the diagnosis of nasal polyps

**DOI:** 10.1186/1710-1492-9-38

**Published:** 2013-10-01

**Authors:** Martha Cottrill, Ruth Ko, Harold L Kim

**Affiliations:** 1 Western University, London, Canada; 2 University of Waterloo, Waterloo, Canada; 3 McMaster University, Hamilton, Canada

## Abstract

**Background:**

Symtomatology of nasal polyps (NP) is relatively non-specific and other nasal conditions that cause nasal may be mistaken for NP. The purpose of this study was to evaluate the accuracy otoscopic (OT) examination in detecting presence of NP by using fiberoptic rhinoscopy (FR) as the gold standard to confirm diagnosis of NP.

**Methods:**

Charts from a single allergy clinic were reviewed for any patient having NP diagnosed by FR. Data collected included gender, age, allergy skin test results, and presence of asthma, aspirin allergy, previous nasal surgeries, intranasal corticosteroid use and leukotriene receptor antagonist use.

**Results:**

The OT examination had 44% sensitivity. In this study, more than half (56%) of patients with NP would have had their NP missed if FR had not been performed in addition to the OT examination.

**Conclusions:**

The standard physical examination procedure is often not sufficient to confirm a diagnosis of NP. FR should be considered in the investigation of patients with rhinitis symptoms.

## Background

Nasal polyps (NP) are typically benign pale-gray semi translucent grape-shaped masses in the nasal cavity that arise from the either the paranasal sinuses or from the mucosa of the nasal cavity [1]. NP are soft, but can cause nasal obstruction, rhinorrhoea, anosmia, postnasal drip, and sometimes facial pain. The prevalence of NP in the general population is about 4% [2]; however NP occur most frequently in patients with asthma and in patients with aspirin sensitivity. Aspirin sensitivity often coincides with NP at a prevalence of approximately 40-80% of patients, and 15% of patients with NP have aspirin sensitivity [3]. The incidence of asthma in patients with NP varies from 20-70% [3]. Along with aspirin sensitivity and asthma, NP are often associated with allergies as well as cystic fibrosis (CF). Patients with NP often have allergies, however most patients with allergies do not have NP (0.5% of 3000 subjects) [3]. Nasal polyposis most often occurs in adults, and the prevalence increases with age [4]. NP rarely occur in children (0.1%) [1], however children with CF are much more likely to have NP (6.7-48%) [5]. Therefore, if NP are found in children, CF should be considered. Symptomatology of NP are relatively non-specific, therefore nasal endoscopy should be performed in order to confirm the diagnosis of NP. Endoscopy will help to exclude other diseases that may be mistaken for NP [3]. Accurate diagnosis of NP is important in order to obtain the best results from treatment. Treatment for NP mainly involves the use of topical or systemic corticosteroids and/or surgery to remove the NP, despite poor prognosis.

Nasal examination is important in assessing patients with NP and other types of rhinitis. Most allergists in Canada examine the nose in a physical examination using an otoscope (OT), but fiberoptic rhinoscopy (FR) is rarely used. The purpose of this study is to determine the sensitivity for identifying NP with OT examination using FR findings as the gold standard.

## Methods

A retrospective chart review was performed in a referral allergist’s practice in Kitchener, Ontario. The allergist has extensive experience with nasal examinations using both the OT and FR for examinations. Participants of the study were consecutive new consultation and follow-up patients that were diagnosed with NP from January 2010 to June 2011. The allergist (HLK) first examined each patient with rhinitis symptoms using an OT with a 4 mm otoscopic speculum followed by FR on all patients. Both nasal passages were examined for NP. The allergist used a standard grading system based on the size of the NP: grade 3 (full blockage of nasal vestibule), grade 2 (blockage of the nasal vestibule beyond the lower edge of the middle turbinate), and grade 1 (smaller than the lower edge of the middle turbinate). Other data were collected from the patients including gender, age, allergy skin test results, and presence of asthma, aspirin allergy, previous nasal surgeries, intranasal corticosteroid use and leukotriene receptor antagonist use.

## Results

**Figure 1:**
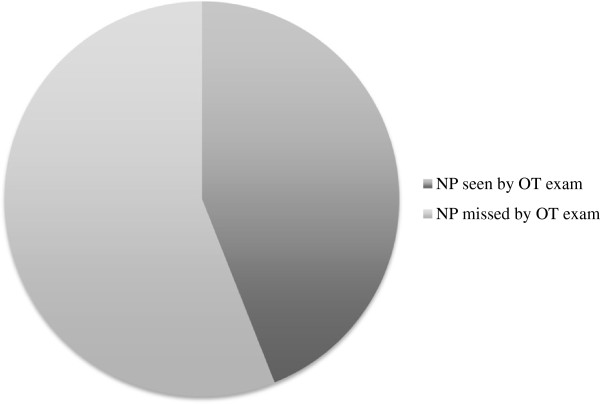
Percentage of nasal polyps (NP) missed by otoscopic (OT) examination compared to fiberoptic rhinoscopy (FR).

A total of 100 patients were identified to have NP with FR in this study. Patients’ ages ranged from 23 to 87 years, and there were 46 males and 54 females. Forty-four patients had NP correctly identified by OT examination (Table 1, Figure 1). Therefore the sensitivity of OT examination was 44% (Sensitivity was calculated by dividing (i) true positive results (as confirmed by FR) by (ii) the sum of true positive and false negative results). For each patient, the largest grade of NP missed by OT was counted. For example, if a patient had grade 1 and grade 3 polyps in each nostril respectively, then only the grade 3 polyps were counted.

**Table 1:** NP presence confirmed by FR

	**PRESENCE**
NP seen with OT	44
NP not seen with OT	56
TOTAL	100

Sensitivity = 44% (Sensitivity = true positives/(true positive + false negative)).

Fifty-six of the 100 patients were misdiagnosed with OT examination. In those patients, the percentages of grades of NP that were missed by OT exam were: 58.9% (33/56) grade 1, 37.5% (21/56) grade 2, and 3.6% (2/56) grade 3 (Table 2).

**Table 2:** Largest grade of polyp missed by OT examination

	**Polyps not seen by OT**
**Grade 1**	33 (58.9%)
**Grade 2**	21(37.5%)
**Grade 3**	2 (3.6%)
**Total**	56

Fifty-two patients with NP had positive skin tests for at least one aeroallergen. Fifty-three patients had asthma (and no ASA allergy), 3 patients had an ASA allergy (and not asthma) and 15 patients with NP had both asthma and ASA allergy. Fifty-eight were using intranasal corticosteroids and 15 were using leukotriene receptor antagonist at the time of assessment. Thirty-seven had previous nasal surgery (Table 3).

**Table 3:** Results for study population, coordinating table information

**Patient characteristics (n = 100)**	
Age range (y)	23–87
Gender (M/F)	46/54
Asthma only (n)	53
ASA sensitivity only (n)	3
ASA sensitivity and asthma (n)	15
Allergic with positive skin tests (n)	52
Nasal steroid (n)	58
Montelukast (n)	15
Previous surgery (n)	37

## Discussion

The sensitivity for identifying NP with OT examination compared to FR findings as the gold standard was assessed in this study. The sensitivity of the OT examination in patients (i.e., per patient) was 44% and therefore, 56% of patients would have been incorrectly diagnosed as not having NP. Smaller NP were more often missed with OT examination than larger NP (Table 2). The high error rate for OT examination is clinically relevant. As far as we are aware, this is the first study to investigate the sensitivity of OT examination compared to FR exam in the diagnosis of NP.

Accurate identification of NP is important due to the altered prognosis with this diagnosis compared to other causes of rhinitis. For example, in a 20-year follow-up study with 41 patients, 85% of patients with NP still suffered from NP even after receiving treatment, and eight of the patients had undergone 11 or more surgeries [3]. Accurate diagnosis of NP may also lead to a different approach to treatment. The chronic nature of NP may lead to ongoing use of topical nasal corticosteroid therapy, systemic corticosteroid therapy and increased likelihood of surgery.

NP have been known to be associated with aspirin sensitivity, asthma, allergy (common aeroallergens), as well as CF. Though the degree to which NP are associated with CF varies; 6.7% to 48% of CF patients have been known to have NP [5], the presence of NP especially in children could indicate CF, and thus correct diagnosis of NP is important [5].

A similar study examined the diagnosis of NP specifically in patients with CF, and found that 25% of NP were missed by physical examination but were seen using FR and 7% of nostrils were misdiagnosed as having NP by physical examination when NP were not in fact present when examined with FR [5].

It is also important to note that many patients with NP will have the significant morbidity of asthma and aspirin allergy: the majority of patients (68%) in our study were diagnosed with asthma. This proportion is relatively high compared to previously published reports, and is likely due to referral bias. As well, a total of 15% of our patients had an ASA allergy. This rate is very close to previously published data [2,6]. These conditions may lead to more severe disease and a poorer prognosis compared to most other types of nasal symptoms. Importantly, some polyploid lesions may be due to neoplasms. If these lesions are not identified, the ultimate clinical outcome may be inferior.

Although the exact cause of NP is unknown, it is often thought that that allergy is a cause of NP [1]. In our study, we identified 58% of patients with allergies to common aeroallergens. Therefore, 42% of patients with NP did not have allergies. This suggests that allergies are not likely a cause of NP for many patients. Furthermore, even including the 58% of patients with allergies, we cannot conclude that allergies directly caused the formation of NP. We must assume that the proportion of patients with allergies may by higher in this study as a result of this study being conducted at an allergist’s practice.

This study had a few potential weaknesses. First, this study was conducted in one center with only one allergist performing both the OT examination and FR examination. Also, the allergist was not blinded to the clinical history for each patient. Hughes and Jones found that 84% of patients with inflammatory nasal conditions were accurately diagnosed based on their history [6]. Finally, this study was performed in a referral allergist’s practice, which could have lead to referral bias. As previously mentioned, the percentage of patients with allergies may have been higher than in the practices of other allergists or otolaryngologists.

In the future, a multi-centre blinded study in which there are multiple examiners should be performed. If a study were performed on all patients with nasal symptoms, the sensitivity, specificity, negative and positive predictive values for OT examination versus FR examinations in the identification of NP could be calculated.

## Conclusion

The OT examination had 44% sensitivity. In this study, more than half (56%) of patients with NP would have had their NP missed if FR had not been performed in addition to the OT examination. This suggests that the standard physical examination procedure is often not sufficient to confirm a diagnosis of NP. Therefore FR should be considered as a key procedure in the investigation of patients with rhinitis symptoms.

## Abbreviations

ASA: Acetyl-salicylic acid; CF: Cystic fibrosis; FR: Fiberoptic rhinoscopy; NP: Nasal polyps; OT: Otoscope.

## Competing interests

The authors have no competing interests to disclose.

## Authors’ contributions

HLK performed the OT and FR examinations and collected all data from the patients. MC and RK analyzed the data and drafted the manuscript. All authors contributed to the study design and manuscript preparation. All authors read and approved the final manuscript.
